# *MONARCC*: a randomised phase II study of panitumumab monotherapy and panitumumab plus 5-fluorouracil as first-line therapy for RAS and BRAF wildtype metastatic colorectal cancer: a study by the Australasian Gastrointestinal Trials Group (AGITG)

**DOI:** 10.1186/s12885-021-08644-4

**Published:** 2021-08-18

**Authors:** Ho Wai Derrick Siu, Niall Tebbutt, Lorraine Chantrill, Chris Karapetis, Christopher Steer, Kate Wilson, David Espinoza, Lisa Bailey, Sonia Yip, Jeff Cuff, Nick Pavlakis, Subotheni Thavaneswaran, Karen Briscoe, Ratnesh Srivastav, Jennifer Shannon, Eva Segelov, Jeannie Tie, Susan Caird, Alessandra Francesconi, Timothy Price, Melanie Wuttke, Rahul Ladwa, Katrin Sjoquist, Matthew Burge

**Affiliations:** 1 NHMRC Clinical Trials Centre, University of Sydney, Sydney, Australia; 2 Camperdown, Australia; 3 Austin Hospital, Melbourne, Australia; 4 Shoalhaven Cancer Care Centre, Nowra, Australia; 5 Flinders Medical Centre, Adelaide, Australia; 6 Border Medical Oncology Research Unit, Albury-Wodonga, Australia; 7 Australasian Gastrointestinal Group (AGITG), Sydney, Australia; 8 Northern Cancer Institute, Sydney, Australia; 9 St Vincent’s Public Hospital, Sydney, Australia; 10 Coffs Harbour Health Campus, Coffs Harbour, Australia; 11 The Tweed Hospital, Tweed Heads, Australia; 12 Nepean Hospital, Sydney, Australia; 13 Monash Medical Centre, Melbourne, Australia; 14 Sunshine Hospital, Melbourne, Australia; 15 Gold Coast University Hospital, Gold Coast, Australia; 16 Sunshine Coast University Hospital, Sunshine Coast, Australia; 17 The Queen Elizabeth Hospital, Adelaide, Australia; 18 Royal Hobart Hospital, Hobart, Australia; 19 Princess Alexandra Hospital, Brisbane, Australia; 20 Royal Brisbane and Women’s Hospital, Brisbane, Australia; 21 The Prince Charles Hospital, Brisbane, Australia

**Keywords:** Metastatic colorectal cancer, Elderly, Older adults, RAS, BRAF, Panitumumab, Cetuximab, Clinical trial

## Abstract

**Background:**

Doublet chemotherapy in combination with a biologic agent has been a standard of care in patients with metastatic colorectal cancer for over a decade. The evidence for a “lighter” treatment approach is limited to mono-chemotherapy plus bevacizumab in the *RAS* unselected population. Anti-EGFR antibodies have activity as monotherapy or in combination with chemotherapy in *RAS* wildtype metastatic colorectal cancer; however their role in first-line treatment in combination with 5-fluorouracil monotherapy or when given alone has not been well studied. MONARCC aims to investigate this approach in an elderly population.

**Methods/design:**

MONARCC is a prospective, open-label, multicentre, non-comparative randomised phase II trial. Eligible patients aged ≥70 with unresectable metastatic, untreated, *RAS*/*BRAF* wildtype metastatic colorectal cancer will be randomised 1:1 to receive panitumumab alone or panitumumab plus infusional 5-fluorouracil. *RAS* and *BRAF* analyses will be performed in local laboratories. Comprehensive Health Assessment and Limited Health Assessments will be performed at baseline and at 16 weeks, respectively, to assess frailty. The Patient Symptom Questionnaire and Overall Treatment Utility are to be undertaken at different timepoints to assess the impact of treatment-related toxicities and quality of life. Treatment will be delivered every 2 weeks until disease progression, unacceptable toxicity (as determined by treating clinician or patient), delay of treatment of more than 6 weeks, or withdrawal of consent. The primary end point is 6-month progression-free survival in both arms. Secondary end points include overall survival, time to treatment failure, objective tumour response rate as defined by RECIST v1.1 and safety (adverse events). Tertiary and correlative endpoints include the feasibility and utility of a comprehensive geriatric assessment, quality of life and biological substudies.

**Discussion:**

MONARCC investigates the activity and tolerability of first-line panitumumab-based treatments with a view to expand on current treatment options while maximising progression-free and overall survival and quality of life in molecularly selected elderly patients with metastatic colorectal cancer.

**Trial registration:**

Australia New Zealand Clinical Trials Registry: ACTRN12618000233224, prospectively registered 14 February 2018.

**Supplementary Information:**

The online version contains supplementary material available at 10.1186/s12885-021-08644-4.

## Background

Doublet chemotherapy (e.g., FOLFOX or FOLFIRI) in combination with a biologic agent (anti-EGFR or anti-VEGF) are established first-line treatments against metastatic colorectal cancer (mCRC) [1, 2]. In patients with *KRAS* exon 2 wildtype mCRC, the use of anti-EGFR antibodies with doublet chemotherapy in the first-line setting has demonstrated superior overall survival (OS) compared with bevacizumab with doublet chemotherapy or doublet chemotherapy alone [1–3]. Subsequent studies have established the benefit of adding anti-EGFR antibodies to first-line doublet therapies is also limited to tumours without mutations in other RAS exons [4, 5]. Therefore, anti-EGFR antibodies are only indicated in patients with no *KRAS* or *NRAS* mutations in exons 2, 3 or 4, which accounts for approximately 40–50% of cases of mCRC. In addition, in patients with *RAS* wildtype disease, the benefit derived from anti-EGFR antibodies is far greater in left-sided than right-sided primary tumours [6, 7]. Approximately 10% of mCRC harbour activating mutations in *BRAF V600E* which are mutually exclusive with *RAS* mutations [8]. Anti-EGFR antibodies have limited, if any activity against *BRAF V600E* mutant cancers, unless combined with a *BRAF* inhibiting agent [9–11]. Therefore, easily determined predictive biomarkers exist to enable selection of patients for treatment with anti-EGFR antibodies.

Patients with mCRC seen in daily practice are often elderly, or have co-morbidities putting them at risk of greater toxicity with standard doublet or triplet regimens than typical populations enrolled onto clinical trials. In addition, some patients have low volume metastases with no or few symptoms. All these patients might be more suited to a “lighter” first-line treatment approach. In elderly patients, number of clinical trials have evaluated such a “lighter” approach in mCRC. AVEX was an open-label phase III randomised control trial that assigned 280 patients aged ≥70 years with untreated, unresectable mCRC to receive capecitabine alone or capecitabine plus bevacizumab. The addition of bevacizumab resulted in an improvement in progression-free survival (PFS) from 5.1 to 9.1 months (HR 0.53; *p* < 0.0001), but OS was not statistically different (HR 0.79; *p* = 0.18) between the two arms. The combination of bevacizumab and capecitabine was well tolerated. However, quality of life and geriatric specific assessments were not performed [12]. The AGITG MAX phase III trial evaluated capecitabine, capecitabine plus bevacizumab, and capecitabine, bevacizumab plus mitomycin as first-line treatment in *RAS* unselected, unresectable treatment naive mCRC. Ninety-nine patients (21%) were identified as age ≥ 75 years in the geriatric subgroup analysis. The PFS benefit derived from the addition of bevacizumab to capecitabine in the elderly population was similar to that in the intention-to-treat population and those < 75 years. There was no signal for increased toxicity in the elderly subgroup [13], supporting the rationale for adding bevacizumab to single-agent chemotherapy in elderly patients.

The effect of age on the efficacy and safety of an anti-EGFR antibody together with doublet chemotherapy were evaluated in subgroup analyses of the PRIME and CRYSTAL trials. Post hoc analysis of the PRIME trial evaluated the effect of age on the efficacy and safety of FOLFOX4 with panitumumab versus FOLFOX4 alone as a first-line treatment in patients with RAS wildtype mCRC. The older age group was defined as those ≥65 years and represented 38% of the participants. Addition of panitumumab to FOLFOX4 was well tolerated and was associated with a trend to increased efficacy in patients aged ≥65 years in terms of PFS (9.7 vs 9.2 months, HR 0.88, 95%CI 0.65–1.19) and OS (26.4 vs 17.4 months HR 0.80, 95%CI: 0.58–1.09). However, there were too few patients aged ≥75 (*n* = 34) to enable firm conclusions to be drawn [14].

Similarly, in the subgroup analysis of the CRYSTAL trial, older age group was defined as ≥65 years and represented about 30% of participants. Addition of cetuximab to FOLFIRI chemotherapy led to a similar improvement in PFS (older group: HR 0.56; 95%CI 0.31–1.03; younger group: HR 0.55; 95%CI 0.38–0.81) and OS (older group: HR 0.91; 95%CI 0.60–1.38; younger group: HR 0.61; 95%CI 0.45–0.82), respectively. However, patients in the older age group had a higher incidence of grade 3–4 treatment-related adverse events compared to the younger age group (77% vs 66.7%, respectively) [15]. The results demonstrated that while elderly patients derived similar survival benefit from anti-EGFR antibodies plus doublet chemotherapy compared to the younger population, this may be associated with higher rates of toxicity.

Studies conducted some years ago have generally found that single-agent chemotherapy results in lower response rates and shorter PFS compared to doublet therapy, but no difference in OS was found when sequencing through second and later-line options was planned [16]. Findings were similar when bevacizumab was added [17]. This lack of evidence of OS benefit with combination chemotherapy has opened the opportunity to investigate treatment de-intensification in the elderly in order to reduce treatment-related toxicity.

The Spanish Cooperative Group for the Treatment of Digestive Tumours (TTD) has conducted a number of studies evaluating the safety and activity of anti-EGFR as monotherapy or in combination with single-agent chemotherapy. In a single-arm phase 2 study, 41 patients aged ≥70 years with untreated mCRC with positive tumour tissue immunohistochemistry for EGFR were treated with single-agent cetuximab. Except for skin toxicities, less than 5% of patients had grade 3 or 4 adverse events. The median time to progression and OS in the intention-to-treat population were 2.9 months and 11.1 months, respectively [18]. A subsequent single-arm phase 2 trial investigated single-agent panitumumab in 33 patients aged ≥70 years with *KRAS* (exon 2) wildtype mCRC. In the subgroup of patients with extended *RAS* wildtype, the median PFS and OS were 7.9 months and 12.3 months, respectively [19]. Cetuximab plus capecitabine was evaluated in a single-arm phase 2 study which recruited 66 *RAS* unselected patients aged ≥70 years. The median PFS and OS of patients with *KRAS* (exon 2) wildtype disease were 8.4 months and 18.8 months, respectively. Cetuximab plus capecitabine also appeared to be less toxic compared with doublet chemotherapy [20]. Even though these studies did not use all the predictive biomarkers we have today, the results suggest that anti-EGFR antibody plus mono-chemotherapy might be a reasonable alternative to more aggressive regimens in the elderly population.

Most recently, the PANDA study was presented in abstract form. This phase 2 study randomised 185 patients aged ≥70 years with untreated wildtype *RAS* and *BRAF* mCRC to receive FOLFOX plus panitumumab or 5-fluorouracil (5-FU) plus panitumumab. The median age of 77 years in both arms is similar to that in other studies investigating an elderly population. The geriatric assessment G8 score was prospectively collected and used as a stratification factor. The primary endpoint of PFS was reached in each arm, at 9.6 months (95% CI 8.8–10.9, *p* < 0.001) and 9.1 months (95% CI 7.7–9.9, *p* < 0.001) in the FOLFOX-panitumumab and the 5-FU-panitumumab arms, respectively. The overall response rate and disease control rate were similar, and the 5-FU plus panitumumab arm had a lower incidence of toxicities. The results demonstrated panitumumab plus mono-chemotherapy is clinically active and well tolerated. However, as chemotherapy was used in both treatment arms, the efficacy of panitumumab monotherapy could not be evaluated [21]. Taken together, these data support further investigation of anti-EGFR antibodies as components of less intensive treatment regimens for elderly patients with untreated *RAS* and *BRAF* wildtype mCRC. A summary of selected studies investigating anti-EGFR antibodies in elderly patients with untreated mCRC is presented in Table 1.

**Table 1:** Selected studies involving anti-EGFR antibodies in elderly patients with mCRC

Study	Design	Population	Treatment	Sample size	Primary endpoint	Key Results
Sastre, 2011 [18]	Prospective single arm, phase II.	Patients aged ≥70 with unselected mCRC	Cetuximab monotherapy	41	Overall response rate (ORR)	ORR 14.6% (95% CI 5.6–29.2)
Sastre, 2012 [20]	Prospective, Single arm, phase II	Patients aged ≥70 with unselected mCRC	Cetuximab plus capecitabine	66	Overall response rate (ORR)	ORR 31.8% (95% CI 20.9–44.4) 29 patients had KRAS wild-type disease: response rate 48.3% (95% CI 29.4–67.5),
Sastre, 2015 [19]	Prospective, Single arm, phase II	Patients aged ≥70 with RAS wild-type mCRC	Panitumumab monotherapy	33	PFS at 6 months	6-month PFS rate 36.4% (95% CI 20.0–52.8)
Kinele, 2018 [22]	Prospective, open label randomised phase II	Patients > 75 years, or patients ≥70 years with at least one adverse factor	Cetuximab monotherapy; cetuximab plus capecitabine	24	PFS at 12 weeks	PFS at 12 weeks: monotherapy arm 55% (95% CI 23–83); combination arm 69%; 95% CI 39–91)
Lonadi, 2019 (abstract) [21]	Prospective, open label randomised phase II	Patients aged ≥70 years with *RAS*-*BRAF* wt mCRC	5FU/LV plus panitumumab; FOLFOX plus panitumumab	185	PFS in both arms	Median PFS: FOLFOX-pan 9.6 m (95% CI 8.8–10.9); 5FU/LV-pan 9.1 m (95% CI 7.7–9.9). Response rate: FOLFOX-pan 65%; 5FU/LV-pan 57%

In this article, we describe the study protocol of MONARCC, a randomised phase II study of panitumumab monotherapy and panitumumab plus 5-FU as first-line therapy for *RAS* and *BRAF* wildtype metastatic colorectal cancer. Importantly, infusional 5-FU was chosen as the partner chemotherapy regimen, given the overlapping toxicities between capecitabine and anti-EGFR antibodies as well as the lack of additional efficacy when anti-EGFR antibodies are added to capecitabine-based regimens [23]. The trial is a collaboration between the Australasian Gastrointestinal Trials Group (AGITG) and the National Health and Medical Research Council (NHMRC) Clinical Trials Centre, University of Sydney, Australia.

## Methods/design

### Aim

MONARCC aims to determine the activity of an anti-EGFR monotherapy in a molecularly selected, hitherto under-investigated, but prevalent elderly patient population. This study investigates a tailored first-line strategy aiming to maximise PFS and OS with acceptable toxicity in a *RAS*/*BRAF* wildtype elderly population suitable for treatment with panitumumab or panitumumab plus infusional 5-fluorouracil (5-FU).

### Design

**Fig. 1:**
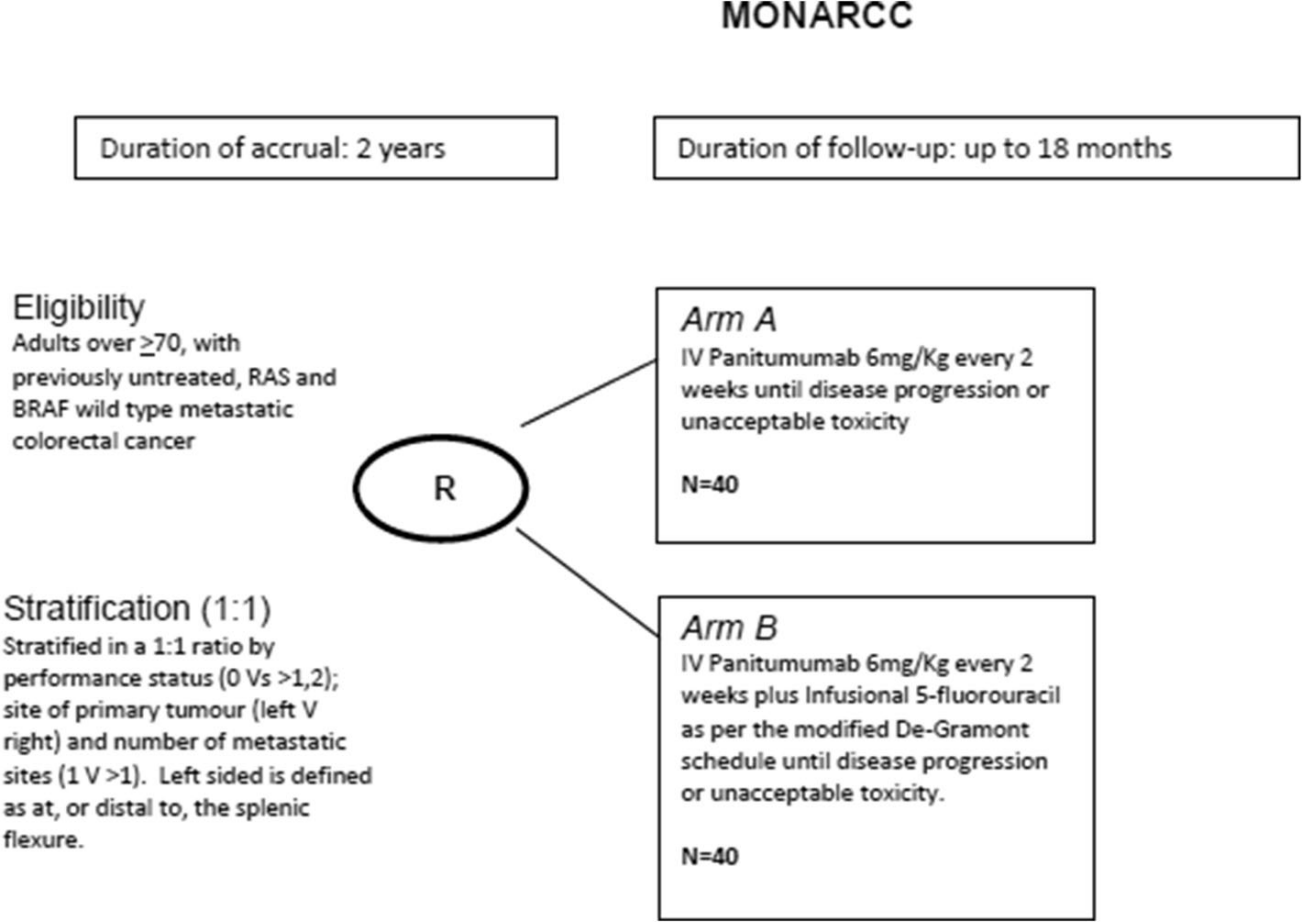
Study schema. Patients are randomised to either panitumumab monotherapy or panitumumab plus infusional 5-FU

*MONARCC* is a prospective, non-comparative randomised phase II, open-label multicentre clinical trial in which patients with histologically confirmed *RAS* and *BRAF* wildtype untreated metastatic colorectal adenocarcinoma are randomised 1:1 ratio to either panitumumab monotherapy (Arm A) or panitumumab plus infusional 5-FU as per the De-Gramont schedule (Arm B). All treatments are to be administered until disease progression, unacceptable toxicity (as determined by treating clinician or patient), treatment delays of longer than 6 weeks, or withdrawal of consent (Fig. 1). Randomisation will be performed centrally using the method of minimisation where patients will be stratified on performance status (Eastern Cooperative Oncology Group [ECOG] 0, 1 vs 2), site of primary tumour (left vs right), number of metastatic sites (1 vs > 1) and treating institution. Left-sided tumour is defined as at, or distal to, the splenic flexure. All patient will receive doxycycline or minocycline 50–100 mg once a day commencing on Cycle 1 Day 1 for a minimum of 6 weeks as a pro-active approach in managing skin toxicity from panitumumab. MONARCC incorporates a number of patient questionnaires and a Comprehensive Health Assessment (CHA) is conducted at baseline. During therapy, patients complete symptom Patient Symptom Questionnaire (PSQ) and Limited Health Assessment Questionnaire (LHA). Overall Treatment Utility (OTU) is also evaluated. This is a novel endpoint using a composite of clinical and radiological response, toxicity, adverse events and patient response when asked to reflect if treatment has been worthwhile. CHA, LHA, PSQ and OTU were originally developed and used in the MRC FOCUS2 study, based on validated and published questionnaires (Additional file 1) [24]. A list of participating centres is provided in Table 2.

**Table 2:** MONARCC participating centres

MONARCC participating sites		Principal investigator
Austin Hospital	Heidelberg	Niall Tebbutt
Border Medical Oncology	Albury	Christopher Steer
Coffs Harbour Base Hospital	Coffs Harbour	Karen Briscoe
Flinders Medical Centre	Bedford Park	Chris Karapetis
The Queen Elizabeth Hospital	Woodville	Timothy Price
Royal Hobart Hospital	Hobart	Melanie Wuttke
Monash Medical Centre Clayton Campus	Clayton	Eva Segelov
The Tweed Hospital	Tweed Heads	Ratnesh Srivastav
Royal Brisbane and Women’s Hospital	Herston	Matthew Burge
Townsville Hospital (Teletrial site)	Townsville	
Hervey Bay Hospital (Teletrial site)	Hervey Bay	
ICON Cancer Centre North Lakes (Teletrial site)	North Lakes	
St Vincent**’**s Hospital (Darlinghurst)	Darlinghurst	Subotheni Thavaneswaran
Sunshine Coast University Hospital	Birtinya	Alessandra Francesconi
Gold Coast University Hospital	Southport	Susan Caird
The Prince Charles Hospital	Chermside	Matthew Burge
Northern Cancer Institute	St Leonards	Nick Pavlakis
Nepean Hospital	Kingswood	Jennifer Shannon
Princess Alexandra Hospital	Woolloongabba	Rahul Ladwa
Sunshine Hospital	St Albans	Jeanne Tie
Shoalhaven Hospital	Nowra	Lorraine Chantrill

### Study endpoints

The primary end point is 6-month PFS in each arm, defined as the interval from date of registration/randomisation to the date of first evidence of disease progression. Disease progression is defined according to RECIST v1.1 [22], as assessed by the investigators.

Secondary objectives of this study are OS, time to treatment failure, objective tumour response rate (according to RECIST v1.1) and safety profile (rates of adverse events per CTCAE v4.03). Time to treatment failure is defined as time from randomisation to recorded discontinuation of treatment for any reason, including disease progression, treatment toxicity and death.

The tertiary and correlative objectives are exploratory comparisons between the treatment arms including, but not limited to, depth of tumour response and early tumour shrinkage, OS and PFS, OTU, feasibility and utility of a comprehensive geriatric assessment using the CHA and LHA questionnaires, quality of life, physical activity measured by an activity tracking device and correlated with other health related parameters, validation of a prognostic nomogram and study associations between clinical outcomes and potential predictive/prognostic biomarkers.

### Study population

Patients aged ≥70 years with ECOG performance status of 0–2, who have cytologically or histologically confirmed previously untreated unresectable mCRC are eligible. *RAS* (*KRAS* exon 2,3 and 4; *NRAS* exon 2 and 3) wildtype, *BRAF* wildtype or non-*V600E BRAF* mutations, as assessed by a local laboratory, are eligible. Other main eligibility criteria include:

1. Measurable disease according to RECIST 1.1.
2. No prior chemotherapy, except for adjuvant chemotherapy given in association with (i) complete resection of primary colon or rectal cancer provided there is no clinical, radiological or biochemical evidence of relapse for at least 6 months after completion of adjuvant treatment and/or (ii) complete resection of limited colorectal metastases to liver and/or lung provided there is no clinical, radiological or biochemical evidence of relapse for at least 6 months after completion of adjuvant treatment.
3. Prior palliative radiotherapy is allowed, provided at least 2 weeks after completion of therapy has elapsed before enrolment, any toxicities have resolved to grade 1 or less.
  - Prior fluoropyrimidine chemotherapy, concurrent with radiation as neoadjuvant treatment for rectal cancer is allowed.
  - Prior radiotherapy, concurrent with radiation sensitising fluoropyrimidines in the setting of metastatic disease is allowed.
4. Adequate organ function defined as follows:
  Bone marrow: ANC (absolute neutrophil count) > 1500/μl, platelets > 75,000/μl, haemoglobin > 8 g/dl. INR (international normalised ratio) and APTT (activated partial thromboplastin time) < 1.5 x ULN (upper limit of normal). Note: patients previously on long-term anticoagulation with warfarin or low molecular weight heparin are eligible.
  Adequate liver function: Albumin > 25 g/l; Total bilirubin < 3 x ULN; AST (aspartate transaminase), ALT (alanine transaminase) and/or ALP (alkaline phosphatase) < 5 x ULN.
  Adequate renal function, creatinine clearance, as measured by the Cockcroft and Gault formula of >30mls/minute.
5. Serum potassium, magnesium and total calcium <grade 2 above or below the institution’s normal limits. Note: total calcium should be corrected for albumin level as per the institution’s usual calculation method.
6. Patient is being treated with non-curative intent. This may be because the disease is anatomically not resectable, that resection is contraindicated for any reason, or the patient refuses resection.

Main exclusion criteria include:

1. History of interstitial lung disease or pulmonary fibrosis.
2. Prior exposure to cetuximab, panitumumab or bevacizumab.
3. Contraindication to study medications.
4. Leptomeningeal disease as the only manifestation of their malignancy.
5. Untreated/active CNS metastases; i.e. progressing, requiring ongoing corticosteroids or anticonvulsants for symptom control. Patients with CNS metastases are eligible if previously successfully treated with surgery and or radiotherapy at least 8 weeks prior to Cycle 1 Day 1, are off all corticosteroids and/or anticonvulsants for at least 4 weeks and imaging within 4 weeks of Cycle 1 Day 1 excludes any progression.
6. Life expectancy of less than 3 months.

### Study procedures

All patients will complete the CHA questionnaire at baseline. Patients enrolled in Arm A and Arm B will receive panitumumab monotherapy 6 mg/kg IV and panitumumab 6 mg/kg IV plus infusional 5-FU (5-FU bolus 400 mg/m^2^, leucovorin 200 mg/m^2^, 5-FU 46-h infusion at 2400 mg/m^2^), respectively. Treatment is delivered every 2 weeks until disease progression, unacceptable toxicities, treatment delay of more than 6 weeks, or withdrawal of consent. For patients needing to cease 5-FU in Arm B, the continuation of panitumumab alone is permitted. Switching the route of administration of 5-FU; e.g., to oral capecitabine, during the study is not permitted. All patients will receive doxycycline or minocycline at 50–100 mg once a day commencing on Cycle 1 Day 1. This will continue for a minimum of 6 weeks but could be continued for longer as deemed appropriate by the investigator. Disease assessment by CT or MRI scan will be performed every 8 weeks until disease progression. The PSQ will be completed every 4 weeks until disease progression. The OTU is to be scored at Weeks 8 and 16 from the PSQ/LHA, with the LHA to be completed once at 16 weeks. Serum CEA is to be assessed every 4 weeks until disease progression.

### Translational analyses

In addition to addressing the clinical questions, the trial also incorporates translational analysis. Archival formalin-fixed paraffin-embedded (FFPE) tissue collection, which is mandatory for study entry, will be used for central review of *RAS*/*BRAF* mutation status and for translational studies. Blood collection are required of all patients for translational endpoints and will be collected at three timepoints: Cycle 1 Day 1, Cycle 3 Day 1 and at 24 weeks. Blood, serum, plasma and tumour specimens will be biobanked. The program will investigate a number of biomarkers identified a priori, which focus on both discovery/ hypothesis generation and validation objectives. No additional tumour samples, other than those obtained for clinical purposes, will be requested.

### Statistical considerations

The primary endpoint, PFS at 6 months in each treatment arm, will be assessed to determine if these protocols are reasonable alternatives to current standard therapy of capecitabine and bevacizumab. The expected PFS rate is approximately 73% in *RAS*/*BRAF* wildtype tumours in prior studies of capecitabine and bevacizumab. Using the method of Mehta-Cain, a total planned sample size of 80 patients, with 40 patients in each group, would include the upper 95% one-sided confidence interval for the proportion of patients who have not progressed at 6 months, including 73% if more than 23 patients are progression-free at 6 months in either group.

The analysis of PFS at 6 months and OS will be estimated using the method of Kaplan-Meier. Analysis of efficacy endpoints will be undertaken in the final analysis, and there is no planned interim analysis.

### Safety

Adverse events will be recorded from the first dose of study treatment until 30 days after cessation of study treatment. The investigator is responsible for ensuring all adverse events observed by the investigator or reported by the trial participants are documented in electronic case report forms (eCRFs). Serious adverse events (SAEs), including suspected unexpected serious adverse reactions occurring during the study must be reported to the sponsor within 24 h of investigational site staff becoming aware of the event according to local procedures. The sponsor is responsible for the medical review of all SAEs and for their notification to the appropriate ethics committees and local authorities.

### Ethics

The study (Protocol v2.0, 12th April 2019) has been approved by the Ethics Review Committee (Royal Prince Alfred Hospital Zone) of the Sydney Local Health District (SLHD), as well individual institutional ethics committees for sites not under SLHD central ethics approval. The study is performed in accordance with the NHMRC Statement on Ethical Conduct in Research Involving Humans 2007, the NHMRC Australian Code for Responsible Conduct of Research 2007, (updated 2015 and as amended from time to time) and the principles laid down by the World Medical Assembly in the Declaration of Helsinki 2008. All participants must provide written informed consent to the study procedures before enrolment in the study.

## Discussion

MONARCC addresses a clinically important question in a prevalent, but under-investigated population that will help inform future practice in elderly patients with *RAS*/*BRAF* wildtype mCRC. Similar to the PANDA trial, MONARCC also incorporates geriatric assessment by prospectively collecting Comprehensive Health and Limited Health Assessment questionnaires, together with physical activity levels measured by activity tracking devices. These aspects of cancer treatment remain under-researched and will add value to the study.

Since the inception of MONARCC in early 2018, other clinical trials have shed light on the use of anti-EGFRs in the elderly population. SAAK 41/10 was a prospective randomised phase II study that evaluated cetuximab monotherapy and cetuximab plus capecitabine as first-line treatment in extended RAS wildtype mCRC; however, the trial was stopped prematurely due to slow accrual. Within the limitations of the small sample size, the authors concluded that upfront cetuximab appeared tolerable and showed promising activity in left-sided tumours [25].

MONARCC is the first study to evaluate the activity of panitumumab monotherapy and panitumumab plus infusional 5-FU in this population. Current accrual (as at 20 November 2020) is 28 patients from 21 sites across Australia. Despite the significance of the clinical question, the recruitment rate of MONARCC has been lower than expected. Challenges to recruitment include a high frequency of *RAS*/*BRAF* mutations, alternative treatment preference such as surgery, multi-agent or oral chemotherapy, and the impact of the COVID-19 pandemic. Strategies to overcome these challenges include setting up tele-trial and satellite sites to expand the study’s reach.

## Supplementary Information

**Additional file 1.**

## Data Availability

Not applicable.

## Abbreviations

5-FU: 5- fluorouracil
AGITG: Australasian Gastro-Intestinal Trials Group
ALP: Alkaline phosphatase
ALT: Alanine transaminase (or alanine aminotransferase)
ANC: Absolute neutrophil count
APTT: Activated partial thromboplastin time
AST: Aspartate transaminase (or aspartate aminotransferase)
CHA: Comprehensive Health Assessment
CNS: Central nervous system
CTCAE: Common Terminology Criteria for Adverse Events
ECOG: Eastern Cooperative Oncology Group
EGFR: Epidermal growth factor receptor
FFPE: Formalin-fixed paraffin-embedded
LHA: Limited Health Assessment Questionnaire
mCRC: Metastatic colorectal cancer
NHMRC CTC: National Health and Medical Research Council Clinical Trials Centre
OS: Overall survival
OUT: Overall Treatment Utility
PFS: Progression- free survival
PSQ: Patient Symptom Questionnaire
QOL: Quality of life
RECIST: Response evaluation criteria in solid tumours
ULN: Upper limit of normal
VEGF: Vascular endothelial growth factor
WT: Wild type
