# Supplementary material for: MONARCC: a randomised phase II study of panitumumab monotherapy and panitumumab plus 5-fluorouracil as first-line therapy for RAS and BRAF wildtype metastatic colorectal cancer: a study by the Australasian Gastrointestinal Trials Group (AGITG)
Source: BMC Cancer. 2021 Aug 18;21:932. doi: 10.1186/s12885-021-08644-4 (PMC8371602; doi:10.1186/s12885-021-08644-4)
Supplement: Supplementary file 1 — Additional file 1. [file 12885_2021_8644_MOESM1_ESM.pdf]

Appendix 1. Baseline Comprehensive Health Assessment
Limited Health Assessment
Patient Symptom Questionnaire
Overall Treatment Utility

Baseline Comprehensive Health Assessment (CHA)

1. Completion by nurse at baseline.

Notes for Research Nurse/Data Manager:

This baseline health assessment is an indispensable part of the trial. It must be completed **after** obtaining the patient's consent, but **before** telephoning the CTC to register and randomise the patient. The CHA is in two parts, (a) a nurse-administered assessment of physical parameters, mental state and medical history, and (b) a patient-completed questionnaire dealing with various aspects of quality of life. **Randomisation will not be performed until both parts of the CHA have been completed.**

CHA Nurse-Administered Section:

For this section of the CHA, you need a quiet, private environment where the patient can answer questions without feeling pressurised or "on trial". Ensure the patient is comfortable and not hungry, thirsty or in need of the toilet or analgesia. Hearing aids, if used, should be working. The patient may have a carer present, but if so ask them not to answer questions on the patient's behalf. If an interpreter is required, use a professional interpreter in preference to a relative.
You will need: a blank sheet of paper; scales and a measure for height and weight; a tape measure for arm circumference. This assessment includes a timed 20-metre walk. For this, a straight 20-metre distance should be marked out, for example in the clinic corridor, and you will need a watch or stopwatch.

Patient initials	Date form completed	Trial N^{o}. (complete after registering)

Section P – Physical tests

P.1) **height in cm:**	P.2) **weight in kg:**	P.3) **right arm circumference in cm** (midway between elbow and shoulder):

P.4) **approximate weight change in past 3 months:**
Ask the patient if they are aware of having lost or gained weight:

☐ don't know

☐ weight loss. If so, how much? Approx …………..kg

☐ weight gain If so, how much? Approx …………..kg

P.5) **timed 20-metre walk.** Use an unobstructed straight 20-metre distance (this should be marked off, for example, in the clinic corridor). Ask the patient to walk as fast as they can without running, starting at the start line and continuing for a few steps beyond the finish line. If they normally use a walking stick or frame, this should be used. Gentle support (e.g. holding elbow) may be given.

Did the patient walk 20 metres? Yes ☐ No ☐
If yes, time taken in seconds: …………………..seconds

If no, give reason: ☐ declined test ☐ unable to walk

Baseline Comprehensive Health Assessment: Completion by nurse at baseline.

Section M (Mini-Mental State Examination)

Introduce this section with something along the lines of: ***"Please don't be offended, but I'd like to ask you a few memory test questions now."***

Then ask the patient the following questions (exact words in **bold**) clearly and score the patient's answers.

M.1) **"What day of the week is it?"** (must be exact to score as correct)	☐ correct ☐ incorrect
M.2) **"What is the date today?"** (must be exact to score as correct)	☐ correct ☐ incorrect
M.3) **"What is the month?"** (must be exact to score as correct)	☐ correct ☐ incorrect
M.4) **"What is the season?"** ("spring" acceptable for Mar – Jun; "summer" for Jun – Sep; "autumn" for Sep – Dec, and "winter" for Dec – Mar inclusive)	☐ correct ☐ incorrect
M.5) **"What is the year?"** (must be exact to score as correct)	☐ correct ☐ incorrect
M.6) **"Where are we now?"** (must be correct but may need clarification)	☐ correct ☐ incorrect
M.7) **"What floor are we on?"** ("upstairs"/"downstairs" is acceptable)	☐ correct ☐ incorrect
M.8) **"In which city are we?"** (accept only the correct answer)	☐ correct ☐ incorrect
M.9) **"In which state are we?"** (accept any correct answer)	☐ correct ☐ incorrect
M.10) **"In which country are we?"** (accept any correct answer)	☐ correct ☐ incorrect
M.11-13) **"I am going to name three objects, After I have finished saying all three, please repeat them. Remember what they are because I am going to ask you to name them again in a few minutes: Apple…. Table…. Penny"** (may repeat up to 5 times)	Apple: ☐ ☐<br>Table: ☐ ☐<br>Penny ☐ ☐<br>correct incorrect
M.14) **"Now please take 7 away from 100"** (only "93" scores correct)	☐ correct ☐ incorrect
M.15) **"Now take 7 away from the number you get"** (score correct if difference is 7, even if previous answer was wrong)	☐ correct ☐ incorrect

Baseline Comprehensive Health Assessment: Completion by nurse at baseline.

M.16-18) "**Now keep going until I ask you to stop"** (score each correct if difference is 7, even if previous answer was wrong)	first: ☐<br>second: ☐<br>third: ☐<br>correct	☐<br>☐<br>☐<br>incorrect
M.19-21) "**What were the three words I asked you to repeat a little while ago?**: (there should be no prompting)	Apple: ☐<br>Table: ☐<br>Penny: ☐<br>correct	☐<br>☐ ☐<br>incorrect
M.22) "**What is this?**: (show a pencil)	☐<br>Correct	☐<br>incorrect
M.23) "**What is this?**" (show a watch)	☐<br>Correct	☐<br>incorrect
M.24)"**I am going to say something and I would like you to repeat it after me. NO IFS, ANDS OR BUTS**: (read this only once)	☐<br>correct	☐<br>incorrect
M.25-27) "**I am going to ask you to carry out some actions. Please listen to the whole command before trying: take this piece of paper, fold it in half and put it on the floor"** (offer a sheet of paper)	Take paper ☐<br>Fold in half: ☐<br>Put on floor: ☐<br>correct	☐<br>☐ ☐<br>incorrect
M.28 "**Please do this**" (close your eyes)	☐<br>correct	☐<br>incorrect
M.29) "**Please write a sentence of your choice on this piece of paper**" (grammar and spelling not important; accept any sentence with a subject (real or implied) and verb. "Help" or "Go away" are acceptable)	☐<br>correct	☐<br>incorrect
M.30) "**Copy this drawing on a piece of paper**" (show the patient the following drawing. To score correct, they must draw two intersection pentagons, and the intersection should be a diamond shape)	☐<br>correct	☐<br>incorrect

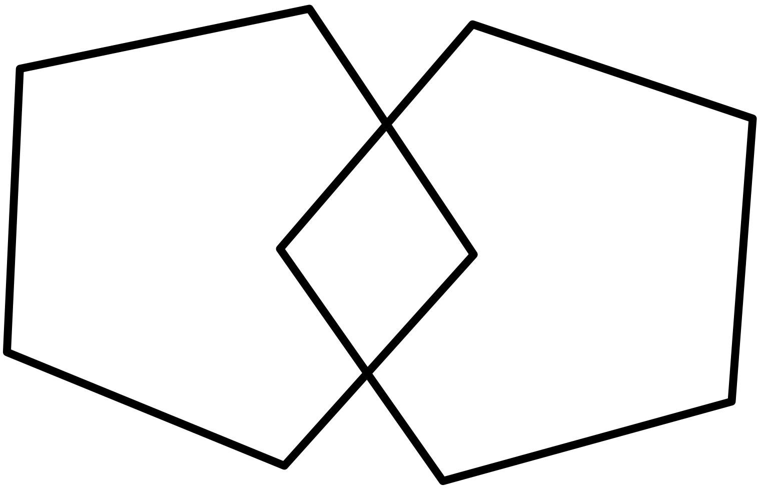

<u>Baseline Comprehensive Health Assessment</u>: Completion by nurse at baseline.

<u>Section C (Charlson Comorbidity Index)</u>

This section is completed from the medical notes, although it is helpful to do so whilst the patient is still present so that you can clarify any missing data (using lay terms).
Record whether there is a past or current history of any of the following medical conditions (if in doubt, consult the doctor responsible):

	Score-points
Myocardial infarct<br>*History of medically documented myocardial infarction*	1
Congestive heart failure<br>*Symptomatic congestive heart failure with response to specific treatment*	1
Peripheral vascular disease<br>*Intermittent claudication, peripheral arterial bypass for insufficiency, gangrene, acute arterial insufficiency, untreated aneurysm (>6cm)*	1
Cerebrovascular disease (except hemiplegia)<br>*History of Transient ischaemic attack or stroke with no or minor sequelae*	1
Dementia<br>*Chronic cognitive deficit*	1
Chronic pulmonary disease<br>*Symptomatic rheumati due to chronic respiratory conditions (including asthma)*	1
Connective tissue disease<br>*Systemic lupus erythematosis, polymyositis, mixed CTD, polymyalgia rheumatic, moderate to severe rheumatoid arthritis.*	1
Ulcer disease<br>*Patients who have required treatment for peptic ulcer disease*	1
Mild liver disease<br>*Cirrhosis without portal hypertension eg, chronic hepatitis*	1
Diabetes (without complications)<br>*Diabetes with medication*	1
Diabetes with end organ damage<br>*Retinopathy, neuropathy, nephropathy*	2
Hemiplegia<br>*Hemiplegia or paraplegia*	2
Moderate or severe renal disease<br>*GFR <30mls/min dialysis, transplantation, uraemic syndrome*	2
2nd Solid tumour (non metastatic)<br>*Initially treated in the last 5 years. Excluding non-melanomatous skin cancer, and in situ cervical ca.*	2
Leukaemia<br>*Any type*	2
Lymphoma, Multiple myeloma<br>*Non Hodgkin's Lymphoma (NHL), Hodgkins, Waldenstrom, multiple myeloma*	2
Moderate or severe liver disease<br>*Cirrhosis with portal hypertension and/or variceal bleeding*	3
2nd Metastatic solid tumour	6
AIDS<br>AIDS and AIDS related complex	6

Baseline Comprehensive Health Assessment (CHA)

2. Completion by patient at baseline.

CHA Patient Questionnaire Section:

This section of the CHA is a patient-completed questionnaire. Please ensure the patient has a relaxed, private environment such as a clinic room, **not a public waiting area**. Ensure they are comfortable and not hungry, thirsty or in need of the toilet or analgesia. The patient may have a carer present, but if so ask them not to answer questions on the patient's behalf. If an interpreter is required, use a professional interpreter rather than a relative, if possible.

Some patients will require help reading or interpreting the questions, or ticking the response boxes. The research nurse or data manager should offer to sit with the patient and help if they wish, but if so should not change the patient's initial "gut reaction" responses. **When the patient has finished, the research nurse or data manager should look through the questionnaire before the patient leaves, check that there are no missing or unclear answers and check that the patient ID and date are complete.**

Dear Patient,

As part of our research into finding the best chemotherapy for your condition, we'd like to ask you some questions about your activities, symptoms and feelings. Would you please go through this questionnaire and, for each question, tick the answer that fits most closely, even if it is not exact. If there are questions you cannot answer, please ask the nurse for help (or, if you would prefer, the nurse can sit with you and read out all the questions).
Thank you!

**Your initials**	**/ /**<br>**Today's Date**	**Trial N^{o}. (Nurse to complete)**

<u>Baseline Comprehensive Health Assessment</u>: Completion by patient at baseline.

First, some questions about what activities you can manage currently:

A.1) Do you walk around outside?	☐ not at all	☐ with help	☐ alone with difficulty	☐ alone easily
A.2) Do you climb stairs?	☐ not at all	☐ with help	☐ alone with difficulty	☐ alone easily
A.3) Do you get in and out of the car?	☐ not at all	☐ with help	☐ alone with difficulty	☐ alone easily
A.4) Do you walk over uneven ground?	☐ not at all	☐ with help	☐ alone with difficulty	☐ alone easily
A.5) Do you cross roads?	☐ not at all	☐ with help	☐ alone with difficulty	☐ alone easily
A.6) Do you travel on public transport?	☐ not at all	☐ with help	☐ alone with difficulty	☐ alone easily
A.8) Do you manage to feed yourself?	☐ not at all	☐ with help	☐ alone with difficulty	☐ alone easily
A.9) Do you manage to make yourself a hot drink?	☐ not at all	☐ with help	☐ alone with difficulty	☐ alone easily
A.10) Do you take hot drinks from one room to another?	☐ not at all	☐ with help	☐ alone with difficulty	☐ alone easily
A.11) Do you do the washing up?	☐ not at all	☐ with help	☐ alone with difficulty	☐ alone easily
A.12) Do you make yourself a hot snack?	☐ not at all	☐ with help	☐ alone with difficulty	☐ alone easily
A.14) Do you manage your own money when you are out?	☐ not at all	☐ with help	☐ alone with difficulty	☐ alone easily
A.15) Do you wash small items of clothing?	☐ not at all	☐ with help	☐ alone with difficulty	☐ alone easily
A.16) Do you do your own shopping?	☐ not at all	☐ with help	☐ alone with difficulty	☐ alone easily
A.17) Do you do a full clothes wash?	☐ not at all	☐ with help	☐ alone with difficulty	☐ alone easily
A.19) Do you read newspapers or books?	☐ not at all	☐ with help	☐ alone with difficulty	☐ alone easily
A.20) Do you use the telephone?	☐ not at all	☐ with help	☐ alone with difficulty	☐ alone easily
A.21) Do you write letters?	☐ not at all	☐ with help	☐ alone with difficulty	☐ alone easily
A.22) Do you go out socially?	☐ not at all	☐ with help	☐ alone with difficulty	☐ alone easily
A.23) Do you manage our own garden?	☐ not at all	☐ with help	☐ alone with difficulty	☐ alone easily
A.24) Do you drive a car?	☐ not at all	☐ with help	☐ alone with difficulty	☐ alone easily

<u>Baseline Comprehensive Health Assessment</u>: Completion by patient at baseline.

E.1) Over the past **6 weeks** how many times has your GP visited you?	☐ not at all	or: How many times?:……………
E.2) Over the past **6 weeks** how many times have you visited your GP?	☐ not at all	or: How many times?:…………….
E.3) Over the past **6 weeks** how often have you been visited by a community nurse?	☐ not at all	or: How many times?:…………….
E.4) Over the past **6 weeks,** have you been visited by a palliative care nurse?	☐ not at all	or: How many times?:…………….

E.5) Which best describes your mobility **today**?	☐ I have no problems walking about	☐ I have some problems walking about	☐ I am confined to bed
E.6) Which best describes your self-care **today**?	☐ I have no problems with self care	☐ I have some problems washing or dressing	☐ I am unable to wash or dress myself
E.7) Which best describes your activities **today**?	☐ I have no problems performing my usual activities	☐ I have some problems performing my usual activities	☐ I am unable to perform my usual activities
E.8) Do you have any pain **today**?	☐ I have no pain or discomfort	☐ I have some pain or discomfort	☐ I have extreme pain or discomfort
E.9) Which best describes your mood **today**?	☐ I am not anxious or depressed	☐ I am moderately anxious or depressed	☐ I am extremely anxious or depressed

<u>Baseline Comprehensive Health Assessment</u>: Completion by patient at baseline.

Now some questions about your symptoms over the past week.

During the past week …				
Q.8) …were you short of breath?	☐ not at all	☐ a little	☐ quite a bit	☐ very much
Q.9) …have you had pain?	☐ not at all	☐ a little	☐ quite a bit	☐ very much
Q.10) …did you need to rest?	☐ not at all	☐ a little	☐ quite a bit	☐ very much
Q.11) …have you had trouble sleeping?	☐ not at all	☐ a little	☐ quite a bit	☐ very much
Q.12) …have you felt weak?	☐ not at all	☐ a little	☐ quite a bit	☐ very much
Q.13) …have you lacked appetite?	☐ not at all	☐ a little	☐ quite a bit	☐ very much
Q.14) …have you felt nauseated?	☐ not at all	☐ a little	☐ quite a bit	☐ very much
Q.15) …have you vomited?	☐ not at all	☐ a little	☐ quite a bit	☐ very much
Q.16) …have you been constipated?	☐ not at all	☐ a little	☐ quite a bit	☐ very much
Q.17) …have you had diarrhoea?	☐ not at all	☐ a little	☐ quite a bit	☐ very much
Q.18) …were you tired?	☐ not at all	☐ a little	☐ quite a bit	☐ very much
Q.31)…have you had a dry and/or sore mouth?	☐ not at all	☐ a little	☐ quite a bit	☐ very much
Q.32)…have you had problems eating or drinking because of a sore mouth?	☐ not at all	☐ a little	☐ quite a bit	☐ very much
Q.33)…have you had soreness or redness of your hands or feet?	☐ not at all	☐ a little	☐ quite a bit	☐ very much
Q.34) …have you had difficulty handling small objects (eg buttons or zips)?	☐ not at all	☐ a little	☐ quite a bit	☐ very much
Q.35) …have you lost any hair?	☐ not at all	☐ a little	☐ quite a bit	☐ very much

Q.36) …if you have a colostomy, have you had trouble with it (soreness of skin, increased frequency, leakage)?	☐ not applicable	☐ not at all	☐ a little	☐ quite a bit	☐ very much

G.1) How was your **overall health** during the past week? *(put a circle round the score)*	**1** Very poor	**2**	**3**	**4**	**5**	**6**	**7** Excellent
G.2) And how was your **overall quality of life** during the past week?	**1** Very poor	**2**	**3**	**4**	**5**	**6**	**7** Excellent

Baseline Comprehensive Health Assessment: Completion by patient at baseline.

Finally, please answer these questions about your feelings. For each statement please mark the box which best describes how you have been feeling over the past week:

H.1) "I feel tense or 'wound up'…"	☐ most of the time	☐ a lot of the time	☐ time-to-time, occasionally	☐ not at all
H.2) "I still enjoy the things I used to enjoy…"	☐ definitely as much	☐ not quite so much	☐ only a little	☐ hardly at all
H.3) "I get a sort of frightened feeling as if something awful is about to happen…"	☐ very definitely and quite badly	☐ yes, but not too badly	☐ a little but it doesn't worry me	☐ not at all
H.4) "I can laugh and see the funny side of things…"	☐ as much as I always could	☐ not quite so much now	☐ definitely not so much now	☐ not at all
H.5) "Worrying thoughts go through my mind…"	☐ a great deal of the time	☐ a lot of the time	☐ time to time but not too often	☐ only occasionally
H.6) "I feel cheerful…"	☐ not at all	☐ not often	☐ sometimes	☐ most of the time
H.7) "I can sit at ease and feel relaxed…"	☐ definitely	☐ usually	☐ not often	☐ not at all
H.8) "I feel as if I am slowed down…"	☐ nearly all the time	☐ very often	☐ sometimes	☐ not at all
H.9) "I get a sort of frightened feeling like 'butterflies' in the stomach…"	☐ not at all	☐ occasionally	☐ quite often	☐ very often
H.10) "I have lost interest in my appearance…"	☐ definitely	☐ I take less care than I should	☐ I may not take quite as much care	☐ I take just as much care as ever
H.11) "I feel restless as if I have to be on the move…"	☐ very much indeed	☐ quite a lot	☐ not very much	☐ not at all
H.12) "I look forward with enjoyment to things…"	☐ as much as I ever did	☐ rather less than I used to	☐ definitely less than I used to	☐ hardly at all
H.13) "I get sudden feelings of panic…"	☐ very often indeed	☐ quite often	☐ not very often	☐ not at all
H.14) "I can enjoy a good book or radio or TV programme…"	☐ often	☐ sometimes	☐ not often	☐ very seldom

Many thanks for helping us by filling in this questionnaire. Please now hand it to the research nurse. You may feel that you wish to discuss some of the issues which it has raised – please feel free to do so.

Patient Symptom Questionnaire (PSQ)

Completion by patient every 4 weeks until disease progression. Do not administer at baseline or week 16 as this questionnaire forms part of the LHA. Q37 and Q38 form part of the Overall Treatment Utility assessment.

All Patient Symptom Questionnaires must be completed prior to the patient seeing the treating clinician on that visit day.

Patient Symptom Questionnaire

Please answer these questions about your symptoms, over the past week….

Q.8) …were you short of breath?	☐ not at all	☐ a little	☐ quite a bit	☐ very much
Q.9) …have you had pain?	☐ not at all	☐ a little	☐ quite a bit	☐ very much
Q.10) …did you need to rest?	☐ not at all	☐ a little	☐ quite a bit	☐ very much
Q.11) …have you had trouble sleeping?	☐ not at all	☐ a little	☐ quite a bit	☐ very much
Q.12) …have you felt weak?	☐ not at all	☐ a little	☐ quite a bit	☐ very much
Q.13) …have you lacked appetite?	☐ not at all	☐ a little	☐ quite a bit	☐ very much
Q.14) …have you felt nauseated?	☐ not at all	☐ a little	☐ quite a bit	☐ very much
Q.15) …have you vomited?	☐ not at all	☐ a little	☐ quite a bit	☐ very much
Q.16) …have you been constipated?	☐ not at all	☐ a little	☐ quite a bit	☐ very much
Q.17) …have you had diarrhoea?	☐ not at all	☐ a little	☐ quite a bit	☐ very much
Q.18) …were you tired?	☐ not at all	☐ a little	☐ quite a bit	☐ very much
Q.31)…have you had a dry and/or sore mouth?	☐ not at all	☐ a little	☐ quite a bit	☐ very much

Patient Symptom Questionnaire: Completion by patient every 4 weeks until disease progression.

Q.32)…have you had problems eating or drinking because of a sore mouth?		☐ not at all	☐ a little	☐ quite a bit	☐ very much
Q.33)…have you had soreness or redness of your hands or feet?		☐ not at all	☐ a little	☐ quite a bit	☐ very much
Q.34) …have you had difficulty handling small objects (eg buttons or zips)?		☐ not at all	☐ a little	☐ quite a bit	☐ very much
Q.35) …have you lost any hair?		☐ not at all	☐ a little	☐ quite a bit	☐ very much
Q.36) If you have a colostomy, have you had trouble with it (soreness of skin, increased frequency, leakage)?	☐ not applicable	☐ not at all	☐ a little	☐ quite a bit	☐ very much
Q.37) …how much has your chemotherapy treatment interfered with your normal daily activities?	☐ not applicable	☐ not at all	☐ a little	☐ quite a bit	☐ very much
Q.38) **Since you started chemotherapy,** how worthwhile do you think your treatment has been?	☐ not applicable	☐ not at all	☐ a little	☐ Quite a bit	☐ very much
G.1) How was your **overall health** during the past week? *(put a circle round the score)*	**1** **2** **3** **4** **5** **6** **7**<br>Very poor … Excellent				
G.2) And how was your **overall quality of life** during the past week?	**1** **2** **3** **4** **5** **6** **7**<br>Very poor … Excellent				

Limited Health Assessment (LHA)

Completion once by nurse and patient ONCE after 16 weeks.

The Limited Health Assessment must be completed prior to the patient seeing the treating clinician on that visit day.

Notes for Research Nurse/Data Manager:

This health assessment, along with the baseline CHA, is an indispensable part of the trial. It is completed once after the first 16 weeks of chemotherapy. These forms will provide important data for the trial. Like the CHA, the LHA is in two sections, (a) a nurse-administered assessment of physical parameters, and (b) a patient-completed questionnaire dealing with various aspects of quality of life.

LHA Nurse-Administered Section:

You will need: scales and a measure for height and weight; a tape measure for arm circumference. This assessment includes a timed 20-metre walk. For this, a straight 20-metre distance should be marked out, for example in the clinic corridor, and you will need a watch or stopwatch.

Patient initials	Date form completed	Trial N^{o}.

Section P – Physical tests

P.1) **height in cm:**	P.2) **weight in kg:**	P.3) **right arm circumference in cm** (midway between elbow and shoulder):

P.4) **approximate weight change in past 3 months:**

Ask the patient if they are aware of having lost or gained weight:

- ☐ don't know
- ☐ weight loss. If so, how much? Approx …………..kg
- ☐ weight gain If so, how much? Approx …………..kg

P.5) **timed 20-metre walk.** Use an unobstructed straight 20-metre distance (this should be marked off, for example, in the clinic corridor). Ask the patient to walk as fast as they can without running, starting at the start line and continuing for a few steps beyond the finish line. If they normally use a walking stick or frame, this should be used. Gentle support (e.g. holding elbow) may be given.

Did the patient walk 20 metres? Yes ☐ No ☐

If yes, time taken in seconds: …………………..seconds

If no, give reason:
- ☐ declined test
- ☐ unable to walk

LHA Patient Questionnaire Section:

This section of the LHA is a patient-completed questionnaire. Please ensure the patient has a relaxed, private environment such as a clinic room, **not a public waiting area**. Ensure they are comfortable and not hungry, thirsty or in need of the toilet or analgesia. The patient may have a carer present, but if so ask them not to answer questions on the patient's behalf. If an interpreter is required, use a professional interpreter rather than a relative, if possible.

Some patients will require help reading or interpreting the questions, or ticking the response boxes. The research nurse or data manager should offer to sit with the patient and help if they wish, but if so should not change the patient's initial "gut reaction" responses. **When the patient has finished, the research nurse or data manager should look through the questionnaire before the patient leaves, check that there are no missing or unclear answers and check that the patient ID and date are complete.**

Dear Patient,

As part of our research into finding the best chemotherapy for your condition, we would like to ask you the same questions that we did at the start of your treatment, about your activities, symptoms and feelings. For each question, tick the answer which fits most closely, even if it is not exact. If there are questions you cannot answer, please ask the nurse for help (or, if you would prefer, the nurse can sit with you and read out all the questions).

Thank you!

**Your initials**	**/ /**<br>**Today's Date**	**Trial N°. (Nurse to complete)**

First, some questions about what activities you can manage currently:

A.1) Do you walk around outside?	☐ not at all	☐ with help	☐ alone with difficulty	☐ alone easily
A.2) Do you climb stairs?	☐ not at all	☐ with help	☐ alone with difficulty	☐ alone easily
A.3) Do you get in and out of the car?	☐ not at all	☐ with help	☐ alone with difficulty	☐ alone easily
A.4) Do you walk over uneven ground?	☐ not at all	☐ with help	☐ alone with difficulty	☐ alone easily
A.5) Do you cross roads?	☐ not at all	☐ with help	☐ alone with difficulty	☐ alone easily
A.6) Do you travel on public transport?	☐ not at all	☐ with help	☐ alone with difficulty	☐ alone easily
A.8) Do you manage to feed yourself?	☐ not at all	☐ with help	☐ alone with difficulty	☐ alone easily
A.9) Do you manage to make yourself a hot drink?	☐ not at all	☐ with help	☐ alone with difficulty	☐ alone easily
A.10) Do you take hot drinks from one room to another?	☐ not at all	☐ with help	☐ alone with difficulty	☐ alone easily

A.11) Do you do the washing up?	☐ not at all	☐ with help	☐ alone with difficulty	☐ alone easily

A.12) Do you make yourself a hot snack?	☐ not at all	☐ with help	☐ alone with difficulty	☐ alone easily
A.14) Do you manage your own money when you are out?	☐ not at all	☐ with help	☐ alone with difficulty	☐ alone easily
A.15) Do you wash small items of clothing?	☐ not at all	☐ with help	☐ alone with difficulty	☐ alone easily
A.16) Do you do your own shopping?	☐ not at all	☐ with help	☐ alone with difficulty	☐ alone easily
A.17) Do you do a full clothes wash?	☐ not at all	☐ with help	☐ alone with difficulty	☐ alone easily
A.19) Do you read newspapers or books?	☐ not at all	☐ with help	☐ alone with difficulty	☐ alone easily
A.20) Do you use the telephone?	☐ not at all	☐ with help	☐ alone with difficulty	☐ alone easily
A.21) Do you write letters?	☐ not at all	☐ with help	☐ alone with difficulty	☐ alone easily
A.22) Do you go out socially?	☐ not at all	☐ with help	☐ alone with difficulty	☐ alone easily
A.23) Do you manage our own garden?	☐ not at all	☐ with help	☐ alone with difficulty	☐ alone easily
A.24) Do you drive a car?	☐ not at all	☐ with help	☐ alone with difficulty	☐ alone easily

E.1) Over the past **6 weeks** how many times has your GP visited you?	☐ not at all	or: How many times?:……………
E.2) Over the past **6 weeks** how many times have you visited your GP?	☐ not at all	or: How many times?:…………….
E.3) Over the past **6 weeks** how often have you been visited by a community nurse?	☐ not at all	or: How many times?:…………….
E.4) Over the past **6 weeks,** have you been visited by a palliative care nurse?	☐ not at all	or: How many times?:…………….

E.5) Which best describes your mobility **today**?	☐ I have no problems walking about	☐ I have some problems walking about	☐ I am confined to bed
E.6) Which best describes your self-care **today**?	☐ I have no problems with self care	☐ I have some problems washing or dressing	☐ I am unable to wash or dress myself
E.7) Which best describes your activities **today**?	☐ I have no problems performing my usual activities	☐ I have some problems performing my usual activities	☐ I am unable to perform my usual activities
E.8) Do you have any pain **today**?	☐ I have no pain or discomfort	☐ I have some pain or discomfort	☐ I have extreme pain or discomfort
E.9) Which best describes your mood **today**?	☐ I am not anxious or depressed	☐ I am moderately anxious or depressed	☐ I am extremely anxious or depressed

Now some questions about your symptoms. Over the past week….

Q.8) …were you short of breath?	☐ not at all	☐ a little	☐ quite a bit	☐ very much

Q.9) …have you had pain?	☐ not at all	☐ a little	☐ quite a bit	☐ very much
Q.10) …did you need to rest?	☐ not at all	☐ a little	☐ quite a bit	☐ very much
Q.11) …have you had trouble sleeping?	☐ not at all	☐ a little	☐ quite a bit	☐ very much
Q.12) …have you felt weak?	☐ not at all	☐ a little	☐ quite a bit	☐ very much
Q.13) …have you lacked appetite?	☐ not at all	☐ a little	☐ quite a bit	☐ very much
Q.14) …have you felt nauseated?	☐ not at all	☐ a little	☐ quite a bit	☐ very much
Q.15) …have you vomited?	☐ not at all	☐ a little	☐ quite a bit	☐ very much
Q.16) …have you been constipated?	☐ not at all	☐ a little	☐ quite a bit	☐ very much
Q.17) …have you had diarrhoea?	☐ not at all	☐ a little	☐ quite a bit	☐ very much
Q.18) …were you tired?	☐ not at all	☐ a little	☐ quite a bit	☐ very much
Q.31)…have you had a dry and/or sore mouth?	☐ not at all	☐ a little	☐ quite a bit	☐ very much
Q.32)…have you had problems eating or drinking because of a sore mouth?	☐ not at all	☐ a little	☐ quite a bit	☐ very much
Q.33)…have you had soreness or redness of your hands or feet?	☐ not at all	☐ a little	☐ quite a bit	☐ very much
Q.34) …have you had difficulty handling small objects (eg buttons or zips)?	☐ not at all	☐ a little	☐ quite a bit	☐ very much
Q.35) …have you lost any hair?	☐ not at all	☐ a little	☐ quite a bit	☐ very much

Q.36) If you have a colostomy, have you had trouble with it (soreness of skin, increased frequency, leakage)?	☐ not applicable	☐ not at all	☐ a little	☐ quite a bit	☐ very much
Q.37) …how much has your chemotherapy treatment interfered with your normal daily activities?	☐ not applicable	☐ not at all	☐ a little	☐ quite a bit	☐ very much
Q.38) **Since you started chemotherapy,** how worthwhile do you think your treatment has been?	☐ not applicable	☐ not at all	☐ a little	☐ quite a bit	☐ very much

G.1) How was your **overall health** during the past week? *(put a circle round the score)*	**1** Very poor	**2**	**3**	**4**	**5**	**6**	**7** Excellent
G.2) And how was your **overall quality of life** during the past week?	**1** Very poor	**2**	**3**	**4**	**5**	**6**	**7** Excellent

Finally, please answer these questions about your feelings. For each statement please mark the box which best describes how you have been feeling over the past week:

H.1) "I feel tense or 'wound up'…"	☐ most of the time	☐ a lot of the time	☐ time-to-time, occasionally	☐ not at all
H.2) "I still enjoy the things I used to enjoy…"	☐ definitely as much	☐ not quite so much	☐ only a little	☐ hardly at all
H.3) "I get a sort of frightened feeling as if something awful is about to happen…"	☐ very definitely and quite badly	☐ yes, but not too badly	☐ a little but it doesn't worry me	☐ not at all
H.4) "I can laugh and see the funny side of things…"	☐ as much as I always could	☐ not quite so much now	☐ definitely not so much now	☐ not at all
H.5) "Worrying thoughts go through my mind…"	☐ a great deal of the time	☐ a lot of the time	☐ time to time but not too often	☐ only occasionally
H.6) "I feel cheerful…"	☐ not at all	☐ not often	☐ sometimes	☐ most of the time
H.7) "I can sit at ease and feel relaxed…"	☐ definitely	☐ usually	☐ not often	☐ not at all
H.8) "I feel as if I am slowed down…"	☐ nearly all the time	☐ very often	☐ sometimes	☐ not at all
H.9) "I get a sort of frightened feeling like 'butterflies' in the stomach…"	☐ not at all	☐ occasionally	☐ quite often	☐ very often
H.10) "I have lost interest in my appearance…"	☐ definitely	☐ I take less care than I should	☐ I may not take quite as much care	☐ I take just as much care as ever
H.11) "I feel restless as if I have to be on the move…"	☐ very much indeed	☐ quite a lot	☐ not very much	☐ not at all
H.12) "I look forward with enjoyment to things…"	☐ as much as I ever did	☐ rather less than I used to	☐ definitely less than I used to	☐ hardly at all
H.13) "I get sudden feelings of panic…"	☐ very often indeed	☐ quite often	☐ not very often	☐ not at all
H.14) "I can enjoy a good book or radio or TV programme…"	☐ often	☐ sometimes	☐ not often	☐ very seldom

Many thanks for helping us by filling in this questionnaire. Please now hand it to the research nurse. You may feel that you wish to discuss some of the issues which it has raised – please feel free to do so.

Overall Treatment Utility (OTU)

Overall Treatment Utility (OTU) is a composite endpoint, originally designed by Matthew Seymour in the UK FOCUS-2 clinical trial[41] which recruited a similar patient population to this study. It is designed to assess the benefit from treatment based on efficacy, toxicity and subjective impact on the patient's quality of life.

OTU can be regarded as asking the physician: "In hindsight, am I glad I gave this treatment to this patient?"; and asking the patient: "In hindsight, am I glad I received this treatment"?

In this study, OTU will be categorised into a 2 point score based on whether the treatment has been clinically effective and tolerable/acceptable (see below).

The patient will complete their Patient Symptom Questionnaire every 4 weeks during treatment and at the end of each of these questionnaires will be asked if they believe their treatment-to date-has been worthwhile.

However OTU will only be formally assessed at 8 weeks and then 16 weeks after randomisation, which is time to coincide with CT scans assessing response/progression.

The scoring of OTU has been modified in this study, with "Intermediate OTU" removed to leave only "**Good OTU"** or "**Poor OTU** "as possible scores for OTU.

1) Definition of clinical efficacy:

Effective:
- **Both** radiologically progression-free (RECIST response or stable disease)
- **And** no clinical deterioration, as assessed by treating consultant

Not effective:
- **Either** radiological progression (RECIST progressive disease)
- **Or** clinical deterioration, as assessed by treating consultant

Clinical deterioration means clear clinical evidence of significant cancer progression that is not evident radiologically. A rise in tumour marker, such as CEA, on its own, would not qualify as clinical deterioration.

2) Definition of tolerability and acceptability:

Tolerable/ acceptable. All of the following:
- No SAE or SUSAR attributed to treatment. However, the SAE or SUSAR event must be felt by the treating clinician to be clinically significant and a threat to the patient's wellbeing.
- No episodes of grade ≥3 non-haematological toxicity. Again, the toxicity event must also be felt by the treating clinician to be of clinical importance and a significant threat to the patient. For example, asymptomatic grade 3 hypomagnesaemia will generally not infer intolerability and a poor OTU.
- Patient response to repeat Q37 ("How much has your treatment interfered with your normal daily activities?") **is not** "Very much" at both OTU assessment timepoints
- Patient response to repeat Q38 ("How worthwhile do you think your treatment has been?") **is not** "Not at all" at both OTU assessment time points

Not tolerable/acceptable. **Any of the following:**

- an SAE or SUSAR clearly attributed to treatment and felt by the investigator to be clinically significant and a threat to the patient's wellbeing.
- an episode of grade ≥3 non-haematological toxicity. Again, the toxicity event must be felt to be of clinical importance and a significant threat to the patient.
- patient response to Q37 ("How much has your treatment interfered with your normal daily activities?") is "Very much" at either OTU assessment timepoint
- patient response to Q38 ("How worthwhile do you think your treatment has been?") is "Not at all"; at that OTU assessment timepoint

Scoring

Good OTU - can only be scored **when all the following apply** at the time of scoring:

- Treatment is effective; and
- Treatment is tolerable/acceptable; and
- Patient is alive.

Poor OTU - can be scored when any of the following apply at the time of scoring:

- Treatment was either not effective OR not tolerable.
- Patient has died at time of assessment.

The matrix below is another way of displaying how to score OUT. As can be seen, the only time OTU can be scored as "good" is when the treatment is both effective and tolerable and the patient is alive, at the time of scoring.

**Score**	**Good**	**Poor**	**Poor**	**Poor**
A: Treatment is effective	x			x
B: Treatment is tolerable / acceptable	x	x		
C: Treatment is not effective		x	x	
D: Treatment is not tolerable/acceptable			x	x
E: Patient has died		x	x	x
